# Zerumbone, a Tropical Ginger Sesquiterpene of *Zingiber officinale* Roscoe, Attenuates α-MSH-Induced Melanogenesis in B16F10 Cells

**DOI:** 10.3390/ijms19103149

**Published:** 2018-10-13

**Authors:** Taek-In Oh, Hye-Jeong Jung, Yoon-Mi Lee, Sujin Lee, Geon-Hee Kim, Sang-Yeon Kan, Hyeji Kang, Taerim Oh, Hyun Myung Ko, Keun-Chang Kwak, Ji-Hong Lim

**Affiliations:** 1 Department of Biomedical Chemistry, College of Biomedical & Health Science, Konkuk University, Chungju 27478, Chungbuk, Korea; dk1050@kku.ac.kr (T.-I.O.); hyevely@kku.ac.kr (H.-J.J.); 201341532@kku.ac.kr (S.L.); rlarsgml4@kku.ac.kr (G.-H.K.); hsb6477@kku.ac.kr (S.-Y.K.); kkang@kku.ac.kr (H.K.); dhxofla555@kku.ac.kr (T.O.); 2 Department of Food Bioscience, College of Biomedical & Health Science, Konkuk University, Chungju 27478, Chungbuk, Korea; yoonmilee@kku.ac.kr; 3 Department of Life Science, College of Science and Technology, Woosuk University, 66 Daehak-ro, Jincheon-eup, Chungcheongbuk-do 27841, Korea; greatmen00@hanmail.com; 4 Department of Research & Development Center, BSB korea Co., Ltd., 66 Daehak-ro, Jincheon-eup, Chungcheongbuk-do 27841, Korea; la_chang@naver.com; 5 Diabetes and Bio-Research Center, Konkuk University, Chungju 27478, Korea

**Keywords:** zerumbone, *Zingiber officinale* roscoe, melanogenesis, MITF, ERK1/2

## Abstract

Zerumbone (ZER), an active constituent of the Zingiberaceae family, has been shown to exhibit several biological activities, such as anti-inflammatory, anti-allergic, anti-microbial, and anti-cancer; however, it has not been studied for anti-melanogenic properties. In the present study, we demonstrate that ZER and *Zingiber officinale* (ZO) extract significantly attenuate melanin accumulation in α-melanocyte-stimulating hormone (α-MSH)-stimulated mouse melanogenic B16F10 cells. Further, to elucidate the molecular mechanism by which ZER suppresses melanin accumulation, we analyzed the expression of melanogenesis-associated transcription factor, microphthalmia-associated transcription factor (MITF), and its target genes, such as *tyrosinase*, *tyrosinase-related protein 1* (*TYRP1*), and *tyrosinase-related protein 2* (*TYRP2*), in B16F10 cells that are stimulated by α-MSH. Here, we found that ZER inhibits the MITF-mediated expression of melanogenic genes upon α-MSH stimulation. Additionally, cells treated with different concentrations of zerumbone and ZO showed increased extracellular signal-regulated kinases 1 and 2 (ERK1/2) phosphorylation, which are involved in the degradation mechanism of MITF. Pharmacological inhibition of ERK1/2 using U0126 sufficiently reversed the anti-melanogenic effect of ZER, suggesting that increased phosphorylation of ERK1/2 is required for its anti-melanogenic activity. Taken together, these results suggest that ZER and ZO extract can be used as active ingredients in skin-whitening cosmetics because of their anti-melanogenic effect.

## 1. Introduction

Melanogenesis, the production of melanin by epidermal melanocytes, is stimulated by the α-melanocyte stimulating hormone (α-MSH) that is secreted from keratinocytes upon exposure to ultraviolet (UV) radiation [1,2]. Stem cell factor (SCF) is another melanogenic factor that strictly controls melanocyte migration, proliferation, and differentiation for maintaining postnatal cutaneous melanogenesis [3]. Microphthalmia-associated transcription factor (MITF), which is a melanogenic transcription factor, is activated through the cAMP-PKA-CREB (cyclic adenosine monophosphate-protein kinase A-cAMP response element binding protein) signaling pathway upon α-MSH stimulation via melanocortin 1 receptor (MC1R) in cytoplasmic membranes of epidermal melanocytes [4]. Several chemicals, such as forskolin and IBMX (3-isobutyl-1-methylxanthine), are known to activate the cAMP-PKA-CREB signaling pathway, leading to the induction of melanogenesis [5]. Several studies have revealed that sustained the activation of extracellular-regulated kinases 1 and 2 (ERK1/2), which is involved in the molecular mechanism of oncogenesis, promotes MITF phosphorylation at Ser73 and its subsequent degradation via ubiquitin-dependent proteolysis [6,7]. Indeed, U0126, a selective ERK1/2 pathway inhibitor, has been reported to increase MITF expression and tyrosinase activity, leading to melanin production [6]. Stabilized and activated MITF increases the expression of melanogenic genes, such as *tyrosinase*, *tyrosinase-related protein 1* (*TYRP1*), and *tyrosinase-related protein 2* (*TYRP2*). Transformation of tyrosine to 3,4-dihydroxyphenylalanine (L-DOPA), which is the initial step of melanogenesis, is catalyzed by tyrosinase. Tyrosinase-related proteins (TRPs) catalyze further oxidation and tautomerization reactions [8]. After melanin synthesis, mature melanin is transported from epidermal melanocytes into the cytoplasm of the basal keratinocytes to protect cells from UV radiation [2].

Abnormally increased melanogenesis causes multiple types of skin disorders, such as skin cancer, chloasma, and freckles [4]; thus, small molecules or natural products that target either the catalytic activity of tyrosinase, or regulators of signaling pathways in melanogenesis, including ERK1/2, or MITF-mediated transcription of melanogenic genes, have been identified as active ingredients to be exploited in the cosmetic industry. Several small molecules, such as kojic acid, arbutin, and niacinamide, are widely used as cosmetic ingredients due to their anti-melanogenic activities. However, reports have shown that kojic acid causes multiple side effects, including cytotoxicity, dermatitis, skin cancer, and hepatocellular carcinoma, because of its genotoxic activity [9]. Although arbutin that was isolated from the bearberry plant has been used to treat hyperpigmentation disorder, recently, its use as a cosmetic ingredient has been restricted due to its several side effects [9]. It has been reported that niacinamide has an anti-melanogenic activity and this activity is carried out by inhibiting melanosome transfer from melanocytes to surrounding keratinocytes [10]. Thus, it has been generally used as a skin whitening compound in the cosmetic industry. In order to overcome the limitations of established skin whitening agents that have several side effects, it is important to develop safe skin whitening ingredients derived from natural sources. For example, α-thujaplicin, linderanolide B, 4-butyl resorcinol, and plumbagin have been identified as anti-melanogenic compounds due to their tyrosinase inhibitory effects [4,11]. Nobiletin, withaferin A, sesamol, and chaetocin exhibit anti-melanogenic properties by modulating signaling intermediates in the melanogenesis pathway [11].

Zerumbone (ZER) is a natural product, which is isolated from the volatile essential oils of plants belonging to the Zingiberaceae family, such as *Zingiber zerumbet* Smith and *Zingiber officinale* Roscoe [12]. Several studies have shown that ZER has a broad range of biological activities, including antimicrobial, antioxidant, anti-diabetic, anticancer, anti-inflammatory, antiallergenic, and anti-angiogenic activities [12]. Interestingly, ZER has been shown to exert a protective effect against ultraviolet A (UVA)-induced oxidative damage in skin keratinocytes, owing to its ability to scavenge reactive oxygen species (ROS) via the activation of nuclear factor-E2-related factor-2 (Nrf2) [1,13]. This suggests that ZER can be used as a functional additive in skin care cosmetics. However, the detailed mechanism of the anti-melanogenic properties of ZER action is yet to be studied.

In the present study, the inhibitory effects of ZER and *Zingiber officinale* (ZO) extract on α-MSH-stimulated melanogenesis, and their underlying mechanisms were studied. Here, we show that ZER significantly suppresses α-MSH induced melanogenesis by upregulating the phosphorylation of ERK1/2 and inhibiting MITF-mediated expression of melanogenic genes.

## 2. Results

### 2.1. Zerumbone (ZER) Suppresses α-MSH Induced Melanogenesis in B16F10 Mouse Melanoma Cells

**Figure 1:**
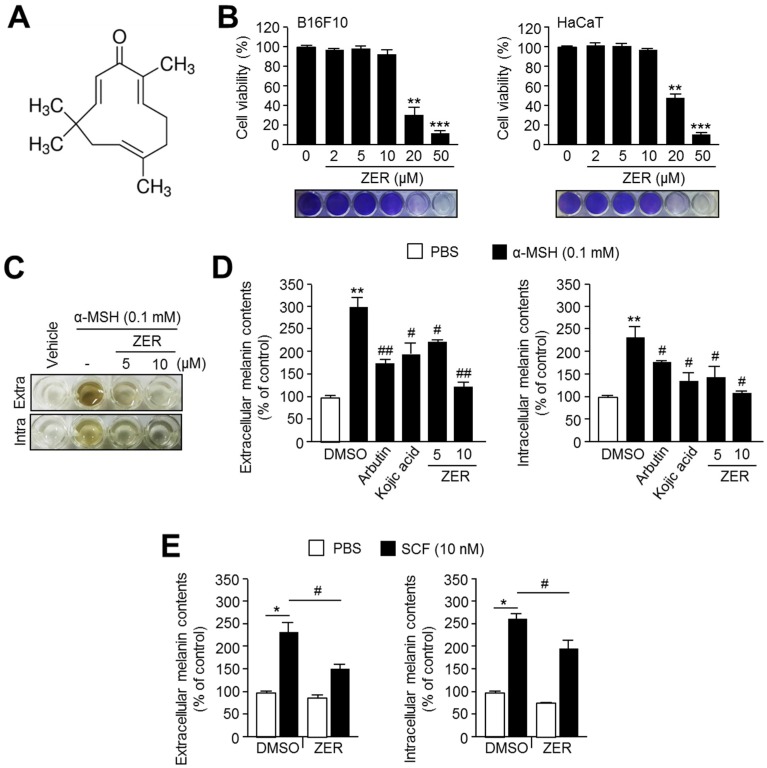
Zerumbone (ZER) suppresses α-melanocytes stimulating hormone (α-MSH)-induced melanin accumulation in mouse melanoma B16F10 cells. (**A**) Chemical structure of zerumbone; (**B**) Cytotoxicity of zerumbone in B16F10 cells. Cells were incubated with 2, 5, 10, 20, and 50 μM of ZER for 72 h. Cell viability was measured using crystal violet staining and stained cell images were shown at bottom panel. Values represent mean ± standard deviations (SD) of three independent experiments performed in triplicate; ** *p* < 0.01 and *** *p* < 0.001; (**C**) Effect of ZER on melanin accumulation and secretion in response to α-MSH stimulation. Cells were pre-treated with ZER (5, 10 μM) for 1 h, and then incubated with α-MSH (0.1 mM) for 3 days; (**D**) Quantitative analysis of anti-melanogenic effect of ZER in compare to arbutin and kojic acid. Cells were pre-treated with zerumbone (5, 10 μM), arbutin (1 mM) or kojic acid (0.2 mM) for 1 h, and then incubated with α-MSH (0.1 mM) for three days. Dimethyl sulfoxide (DMSO) was used as vehicle. Intra or extracellular melanin contents were measured as described in method section. Values represent mean ± SD of three independent experiments performed in duplicate; ** *p* < 0.01 versus vehicle; and # *p* < 0.05 and ## *p* < 0.01 versus α-MSH treated sample; (**E**) Anti-melanogenic effect of ZER in G361 human melanoma cells. Cells were pre-treated with ZER (10 μM) for 1 h, and then incubated in the absence or presence of 10 nM stem cell factor (SCF) for three days. DMSO was used as vehicle. Values represent mean ± SD of three independent experiments performed in duplicate; * *p* < 0.05 and # *p* < 0.05.

In order to study the anti-melanogenic effect of zerumbone (ZER), we initially evaluated its cytotoxicity in both B16F10 and HaCaT cells. The chemical structure of ZER is shown in Figure 1A. It was observed that ZER at concentrations above 20 μM exhibited a strong cytotoxic effect, whereas at those below 20 μM, ZER did not show cytotoxicity in both cell lines (Figure 1B). Next, we investigated the inhibitory effects of ZER on α-melanocytes stimulating hormone (α-MSH)-induced melanin accumulation and secretion in B16F10 cells. ZER was shown to strongly suppress α-MSH induced intracellular accumulation of melanin and its secretion into the cultured medium (Figure 1C,D). Moreover, we found that ZER attenuates melanogenesis more effectively than 1 mM arbutin or 0.2 mM kojic acid, the well-known active constituents of skin-whitening cosmetics (Figure 1D). To elucidate whether ZER is sufficient to suppress melanogenesis in human cells, we used melanin-producing G361 human melanoma cells. As shown in Figure 1E, ZER significantly decreases stem cell factor (SCF)-induced extra- and intracellular melanin contents. These results confirm the anti-melanogenic activity of ZER in melanogenic mouse B16F10 and human G361cells.

### 2.2. Zerumbone Suppresses Gene Expression of Melanogenesis Transcription Factor, MITF, and Its Target Genes in G361 Human Melanoma Cells

Endothelin-1 and SCF signaling have been reported to play essential roles in melanogenesis in human melanocytes and in several subtypes of melanoma [3,14,15,16]. In this study, we found that ZER suppresses melanogenesis upon melanogenic stimuli, α-MSH and SCF, in mouse B16F10 and human G361 melanoma cells (Figure 1).

**Figure 2:**
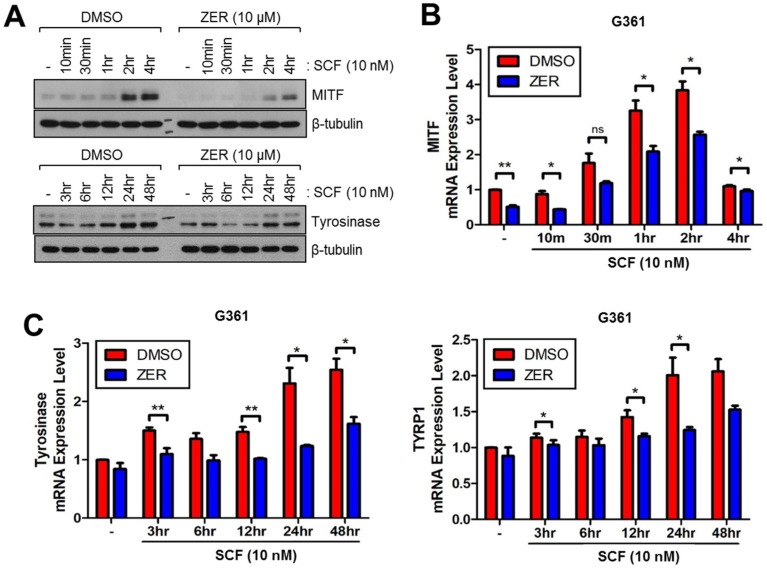
Zerumbone (ZER) decreases expression of melanogenesis-related genes and proteins upon SCF stimulation in G361 human melanoma cells. (**A**) Protein expression levels of microphthalmia-associated transcription factor (MITF) and tyrosinase in presence and absence of ZER. Cells were pre-treated with 10 μM ZER for 1 h, and then incubated with 10 nM SCF for different time intervals as indicated; (**B**) mRNA expression levels of MITF in presence and absence of ZER upon SCF stimulation. Cells were pre-treated with ZER (10 μM) for 1 h, followed by incubation with 10 nM SCF for different time intervals as indicated. Values represent mean ± SD of two independent experiments performed in triplicate; * *p* < 0.05 and ** *p* < 0.01.; (**C**) Tyrosinase and TYRP1 mRNA levels in the absence or presence of ZER upon SCF stimulation. ZER (10 μM) was pre-treated for 1 h and then cells were further incubated with 10 nM of SCF for indicated time. Values represent mean ± SD of two independent experiments performed in triplicate; * *p* < 0.05 and ** *p* < 0.01.

Thus, we measured alterations in the expression levels of melanogenesis-related genes and proteins upon SCF stimulation in G361 human melanoma cells. We found that ZER attenuates SCF-induced MITF and tyrosinase protein expression 2–4 h and 24–48 h after SCF stimulation, respectively (Figure 2A). In addition, gene expression of melanogenesis-related genes, such as *MITF*, *tyrosinase*, and *tyrosinase-related protein 1* (*TYRP1*) was suppressed in ZER-treated G361 cells (Figure 2B,C). These results revealed the peak time for the expression of melanogenesis-related genes upon SCF stimulation; maximal *MITF* mRNA induction was observed within 1–2 h, and mRNA levels of *tyrosinase* and *TYRP1* were observed to reach their maximum 24–48 h after SCF stimulation.

### 2.3. Zerumbone Suppresses Melanogenic Genes and Enzymes Expression in Mouse B16F10 Cells

**Figure 3:**
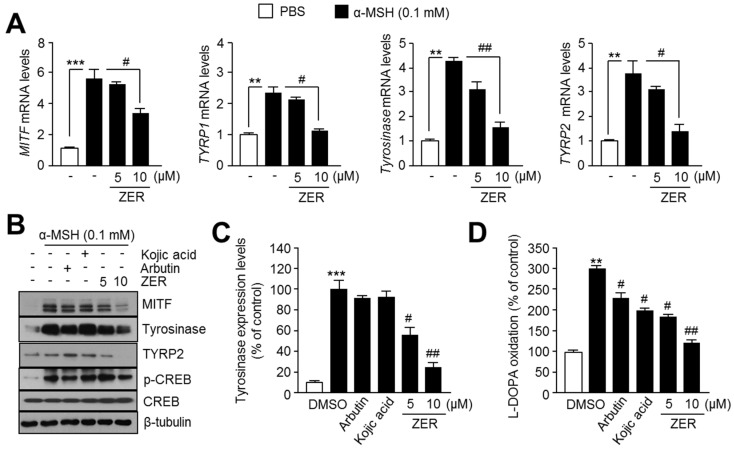
Zerumbone (ZER) decreases expression of melanogenesis-associated transcription factor, MITF, and its target genes upon α-MSH stimulation. (**A**) Gene expression of MITF and its target genes in presence and absence (−) of ZER. Cells were incubated with 5 and 10 μM ZER, followed by treatment with α-MSH for 2 h (for MITF detection) or 48 h (for tyrosinase, TYRP1 and TYRP2 detection). mRNA levels were measured by quantitative real time-PCR as described in “Materials and Methods” section. Values represent mean ± SD of two independent experiments performed in triplicate; ** *p* < 0.01 and *** *p* < 0.001 versus vehicle; and # *p* < 0.05 and ## *p* < 0.01 versus α-MSH treated sample; (**B**) Protein expression of MITF and its target in presence (+) and absence (−) of ZER. Cells were pretreated with ZER (5 or 10 μM), arbutin (1 mM), and kojic acid (0.2 mM) for 1 h, followed by exposure to α-MSH (0.1 mM) for 2 h (for p-CREB and CREB detection), 4 h (for MITF detection), and 48 h (for tyrosinase, TYRP2, and β-tubulin detection), based on the time at which the expression of the different proteins was maximum. Protein expression was measured by immunoblot analysis as described in “Materials and Methods” section; (**C**) Quantitative analysis of tyrosinase protein expression level. Protein expressions were quantified using Image J 1.49v software (National Institutes of Health, Bethesda, MD, USA). Values represent the mean ± SD of three independent experiments performed; *** *p* < 0.001 versus vehicle; and # *p* < 0.05 and ## *p* < 0.01 versus α-MSH treated sample; and, (**D**) Cellular tyrosinase activity. Cellular tyrosinase activity was determined by L-DOPA oxidation to dopachrome, and oxidized L-DOPA was measured at 475 nm using an absorbance reader. Detailed procedure for cellular tyrosinase activity assay has been described in “Materials and Methods” section. Cells were incubated with ZER (5, 10 μM), arbutin (1 mM), and kojic acid (0.2 mM), for 48 h upon α-MSH (0.1 mM) treatment. Values represent mean ± SD of three independent experiments performed in duplicate; ** *p* < 0.01 versus vehicle; and # *p* < 0.05 and ## *p* < 0.01 versus α-MSH treated sample.

To investigate the molecular mechanism by which ZER suppresses melanogenesis, we measured gene expression of melanogenesis transcription factor, *MITF*, and its target genes, such as *tyrosinase-related protein 1* (*TYRP1*), *tyrosinase*, and *tyrosinase-related protein 2* (*TYRP2*) in the absence or presence of ZER. Since the peak time for melanogenesis-related genes expression upon SCF and α-MSH stimulation is known for human G361 (Figure 2) and mouse B16F10 melanoma cells [4], respectively, we further measured the expression of *MITF*, *tyrosinase*, *TYRP1*, and *TYRP2* in ZER treated mouse B16F10 melanoma cells after 2 h or 48 h of incubation with α-MSH. Figure 3A shows that ZER is sufficient to attenuate α-MSH-induced *MITF*, *TYRP1*, *TYRP2*, and *tyrosinase* expression in B16F10 cells. Similarly, MITF, tyrosinase, and TYRP2 protein expression levels were decreased upon ZER treatment. Protein kinase A (PKA)-mediated phosphorylation of CREB is a major signaling pathway increasing MITF at the transcriptional level upon α-MSH stimulation [4]. Thus, here, we analyzed whether decreased phosphorylation of CREB by ZER could mediate suppression of MITF. We found that α-MSH-induced CREB phosphorylation was not affected by ZER treatment, suggesting that ZER suppresses α-MSH-induced MITF expression independent of PKA-CREB signaling pathway axis (Figure 3B). Because tyrosinase is a rate-limiting enzyme, regulating melanin synthesis [2,4,11], we further analyzed intracellular tyrosinase protein levels and its enzymatic activity upon arbutin, kojic acid, and ZER treatment. Figure 3C shows that ZER sufficiently reduces α-MSH-induced tyrosinase protein levels in B16F10 cells. In addition, Figure 3D reveals that 10 μM ZER more effectively suppresses L-DOPA oxidation than tyrosinase inhibitors, arbutin, and kojic acid, suggesting that the oxidation of L-DOPA to dopaquinone through enzymatic activity of tyrosinase is suppressed in ZER-treated cells. These results demonstrate that ZER attenuates α-MSH-mediated melanogenesis by suppressing gene expression of MITF, a melanogenesis-associated transcription factor, and its target genes.

### 2.4. Anti-Melanogenic Effect of Zerumbone Is through Phosphorylation of ERK1/2

**Figure 4:**
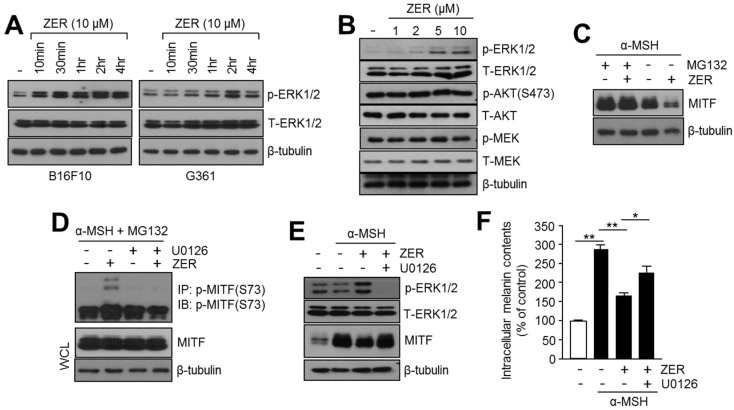
Activation of ERK1/2 is involved in the anti-melanogenic effect of zerumbone (ZER). (**A**) Phosphorylation of ERK1/2 upon ZER treatment. B16F10 and G361 cells were incubated with 10 μM ZER for different time intervals as indicated. Protein expression levels of p-ERK1/2, total-ERK1/2, and β-tubulin (loading control) were analyzed by immunoblotting; (**B**) Alterations in signaling pathways upon ZER treatment. B16F10 cells were treated with different concentrations of ZER for 1 h, and then the total protein was analyzed by immunoblotting to determine activation of ERK1/2, AKT, and MEK as described in the “Materials and Methods” section; (**C**) Proteasome inhibitor, MG132, blocks ZER-mediated reduction of MITF. B16F10 cells were pre-treated with 10 μM ZER for 1 h, and then cells were incubated in the absence (−) or presence (+) of MG132 (20 μM) for 6 h; (**D**) ZER increases phosphorylation of MITF. B16F10 cells were pre-treated with 20 μM MG132 for 3 h, and then cells were incubated in the absence (−) or presence (+) of ZER (10 μM) and U0126 (10 μM) for 1 h. Phosphorylated MITF was determined by immunoprecipitation and immunoblotting; (**E**) Immunoblot analysis of MITF and p-ERK1/2 in absence (−) or presence (+) of ZER or U0126, a selective inhibitor of MAPK. B16F10 cells were treated with U0126 (10 μM) and ZER (10 μM), and were subsequently incubated with α-MSH (0.1 mM) for 1 h (for p-ERK1/2 and T-ERK1/2 detection) and 4 h (for MITF and β-tubulin detection); and, (**F**) Estimation of intracellular melanin content in absence and presence of U0126 and ZER, in response to α-MSH stimulation. Cells were cultured with ZER (10 μM) and U0126 (10 μM), and incubated with α-MSH (0.1 mM) for three days, as indicated. Detailed procedure for melanin content analysis is described in “Materials and Methods” section. Values represent mean ± SD of three independent experiments performed in triplicate; * *p* < 0.05 and ** *p* < 0.01.

Activation of protein kinase B (AKT) and extracellular signal-regulated kinases (ERK1/2) by growth factors or melanogenic stimuli is known to suppress melanogenesis through the decreased expression of MITF and its target genes [11]. Phosphorylation of MITF at Ser73 and Ser409 in response to the ERK1/2 signaling pathway, promotes its proteasome-dependent degradation [17,18]. Therefore, we investigated whether ZER regulates ERK1/2 phosphorylation in B16F10 mouse and G361 human melanoma cells. Increased phosphorylation of ERK1/2 (p-ERK1/2) but not total ERK1/2 (T-ERK1/2) was observed upon ZER treatment in a time- and dose-dependent manner (Figure 4A,B). Interestingly, the phosphorylation of ERK1/2 was observed to be rapidly increased within 10–30 min and 1–2 h post ZER treatment in mouse B16F10 and human G361 melanoma cells (Figure 4A). However, the phosphorylation of AKT and MEK was not observed upon ZER treatment, suggesting that ZER-induced ERK1/2 phosphorylation may suppress MITF (Figure 4B). Because ERK1/2 phosphorylation promotes the phosphorylation and proteasomal degradation of MITF [17,18], we investigated whether proteasome inhibitor prevents reduction of MITF by ZER. Suppression of MITF by ZER was not observed in the presence of MG132, confirming that ZER decreases MITF expression through proteasomal degradation (Figure 4C). Next, we investigated whether ZER phosphorylates MITF at Ser73, which is the target residue of ERK1/2. To detect phosphorylated MITF, cells were exposed to α-MSH and MG132. Here, we found that phosphorylated MITF (Ser73) was significantly increased by ZER in the presence of α-MSH and MG132 (Figure 4D). U0126, a selective mitogen-activated protein kinase (MAPK) inhibitor [19], abolished ZER-induced MITF phosphorylation (Figure 4D). Moreover, total MITF protein levels in whole cell lysates (WCL) were not altered by ZER and U0126 in the presence of MG132 (Figure 4D). These results indicate that ERK1/2-mediated MITF (Ser73) phosphorylation and proteasomal degradation is a critical mechanism in ZER-mediated suppression of MITF. In addition, Figure 4E shows that U0126 significantly restored reduced MITF protein level in ZER treated cells. Consistently, decreased intracellular melanin content by ZER was restored by U0126 to approximately 40% (Figure 4F), suggesting that the activation of ERK1/2 is required for the anti-melanogenic effect of ZER.

### 2.5. Anti-Melanogenic Effect of Zingiber Officinale (ZO) Extracts

**Figure 5:**
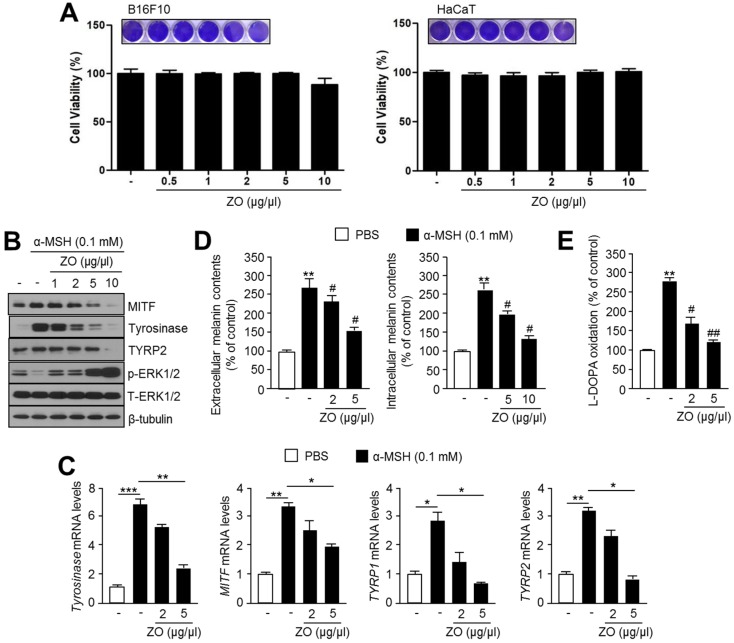
Anti-melanogenic effect of *Zingiber officinale* (ZO) extract is through activation of ERK1/2-mediated suppression of MITF and its target genes expression. (**A**) Cytotoxicity of ZO extract in B16F10 and HaCaT cells. Cells were incubated with 0.5, 1, 2, 5, and 10 μg/μL of ZO extract for 72 h. Cell viability was measured using crystal violet staining, images of which were shown in the upper panel. Values represent mean ± standard deviations (SD) of three independent experiments performed in triplicate; (**B**) Immunoblot analysis of MITF, tyrosinase, and p-ERK1/2 in response to ZO extract in α-MSH-stimulated cells. Cells were pre-treated with different concentrations of ZO extract (1, 2, 5 and 10 μg/μL), followed by incubation with α-MSH (0.1 mM) for 1 h (for p-ERK1/2 and T-ERK1/2 detection), 4 h (for MITF detection), or 48 h (for tyrosinase, TYRP2, and β-tubulin detection). Protein levels were measured by immunoblotting; (**C**) Effect of ZO extract on mRNA expression of MITF and its downstream genes tyrosinase, TYRP-1 and TYRP-2, upon α-MSH stimulation. Cells were pre-treated with ZO extract for 1 h, and then incubated with α-MSH for 2 h (for MITF detection) and 48 h (for tyrosinase, TYRP1, and TYRP2 detection). mRNA levels were measured by quantitative real time-PCR as described in “Materials and Methods” section. Values represent mean ± SD of two independent experiments performed in triplicate; * *p* < 0.05, ** *p* < 0.01 and *** *p* < 0.001; (**D**) Effect of ZO extract on melanin accumulation and secretion in response to α-MSH stimulation. Cells were cultured with ZO extract (2 or 5 μg/μL), followed by incubation with α-MSH (0.1 mM) for 3 days. Intracellular and extracellular melanin content were estimated and analyzed as described in “Materials and Methods” section. Values represent mean ± SD of three independent experiments performed in duplicate; ** *p* < 0.01 versus vehicle; and # *p* < 0.05 versus α-MSH treated sample; and, (**E**) Effect of ZO extract on L-DOPA oxidation activity of cellular tyrosinase using cell lysates. Cells were incubated with ZO extract (2 or 5 μg/μL) for 48 h, followed by incubation with α-MSH (0.1 mM). Values represent mean ± SD of three independent experiments performed in duplicate; ** *p* < 0.01 versus vehicle; and # *p* < 0.05 and ## *p* < 0.01 versus α-MSH treated sample.

ZER is a phytochemical derived from several plant species of the Zingiberaceae family, such as *Zingiber zerumbet* and *Zingiber officinale* [12]. Thus, we initially measured the cell viability in the absence or presence of ZO extract in B16F10 and HaCaT cells. Figure 5A shows that cell viability was not altered by ZO extract treatment. We further analyzed the anti-melanogenic effects of *Zingiber officinale* (ZO) extract. Because the expression of MITF and its target melanogenic genes were decreased by ZER through activated ERK1/2 signaling pathway (Figure 2 and Figure 3), we checked whether ZO extract suppresses α-MSH-induced MITF and its target melanogenic proteins expression in B16F10 cells. Similar to the anti-melanogenic effect of ZER, α-MSH-induced MITF, tyrosinase, and TYRP2 were significantly decreased by the ZO extract in a dose-dependent manner (Figure 5B). Interestingly, ERK1/2 phosphorylation was also increased in ZO extract treated cells (Figure 5B). Expression of *MITF* and its target genes, such as *tyrosinase*, *TYRP1*, and *TYRP2*, were significantly decreased upon 5 μg/μL ZO extract treatment (Figure 5C). Moreover, both extracellular and intracellular melanin content were significantly reduced to approximately 40% upon 5 μg/μL ZO extract treatment (Figure 5D). In addition, decreased tyrosinase activity was observed in cells that were treated with ZO extract (Figure 5E). These results suggest that *Zingiber officinale* (ZO) extract suppresses melanogenesis by attenuating MITF-mediated melanogenic genes expression.

## 3. Discussion

Although ZER exhibits a variety of biological functions, including anti-inflammatory, anticancer, and antimicrobial activities, its anti-melanogenic properties have not been reported [12]. In the current study, we demonstrated for the first time that *Zingiber officinale* (ZO) extract and its active ingredient, ZER, have strong inhibitory effect on α-melanocytes stimulating hormone (α-MSH)-induced melanogenesis.

Abnormally increased melanogenesis caused by ultraviolet (UV) irradiation, inflammatory cytokines, and hormonal signaling, is closely associated with pigmentation disorders, such as chloasma and freckles [4]. Upon UV exposure, keratinocytes secrete α-MSH, which stimulates melanin biogenesis in epidermal melanocytes [1]. In the present study, we demonstrated that methanolic root extract of *Zingiber officinale* (ZO) and ZER strongly suppresses α-MSH-induced melanin accumulation. Comparison of inhibitory effects of arbutin, which is a well-known anti-melanogenic chemical, and ZER on melanin accumulation showed that ZER at 10 μM concentration exhibits approximately 40% stronger anti-melanogenic effect than arbutin in α-MSH-treated B16F10 mouse melanogenic cells.

Several biochemical studies have shown that the essential oil of *Zingiber zerumbet* rhizomes contains a large amount of ZER, accounting for approximately 13–70% of the plant ZER content. However, small amounts of ZER are present in *Zingiber officinale* as well [20]. Interestingly, previous reports have shown that *Zingiber zerumbet* cultivated in South India contains 76.3 to 84.8% of ZER. However, a silviculture farm in India has shown that 1.81% ZER content was found in the rhizome, 0.16% in the root, and 0.09% in the leaf of *Zingiber zerumbet* [12]. Therefore, these backgrounds suggest that the differences in ZER content of *Zingiber zerumbet* may not be correlated with geographic or ecological variations, but instead are because of differences in ZER chemotype [12]. If so, why does ZO have anti-melanogenic activity? A possibility could be suggested that other active components of ZO, which are different from ZER, may suppress melanogenesis. Indeed, a previous report has shown that essential oil of *Zingiber officinale* rhizome contains numerous bioactive components, such as α-pinene, valencene, and zingiberene [21]. Moreover, anti-melanogenic effects of α-pinene and valencene have also been observed in B16F10 mouse melanoma cells [22,23]. In addition, melanogenesis-inhibitory effect of [6]-shogaol, the major shogaol in *Zingiber officinale* rhizomes, has been observed to be through acceleration of ERK1/2-mediated MITF degradation [24]. These previous reports support our result that multiple types of active components of ZO as well as ZER have anti-melanogenic activity.

**Figure 6:**
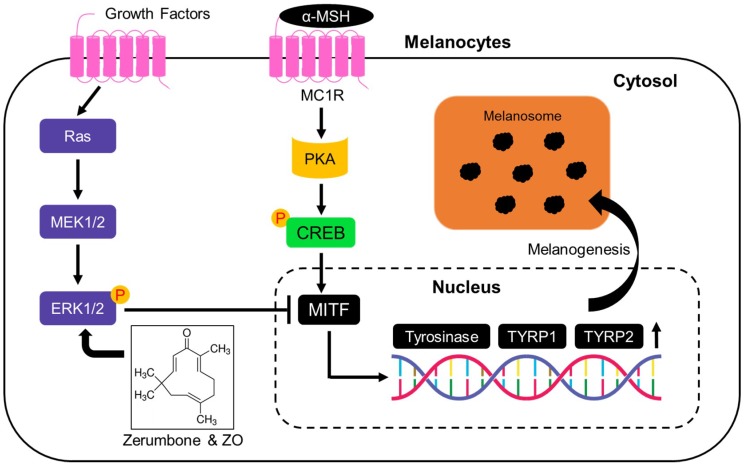
Proposed molecular mechanism of zerumbone (ZER) for attenuation of α-MSH-induced melanogenesis. Black T bar indicates suppression and black arrows indicate activation signaling. α-MSH stimulates MITF mRNA expression through PKA-CREB axis-mediated transcription. Increased ERK1/2 phosphorylation by ZER suppresses MITF. Suppression of MITF by ZER-mediated ERK1/2 phosphorylation down-regulates transcription of genes, such as tyrosinase, TYRP1, and TYRP2, encoding melanogenic enzymes and results in inhibition of melanogenesis. α-MSH: alpha-melanocyte stimulating hormone; MC1R: melanocortin 1 receptor; PKA: protein kinase A; CREB: cAMP response element binding protein; MITF: micropthalmia-associated transcription factor; TYRP1: tyrosinase-related protein 1; TYRP2: tyrosinase-related protein 2; MEK1/2: mitogen-activated protein kinase kinases; and, ERK1/2: extracellular signal-regulated kinases.

Microphthalmia-associated transcription factor (MITF) is pivotal factor for melanogenesis by facilitating transcription of genes, such as *tyrosinase*, *tyrosinase-related protein 1* (*TYRP1*), and *tyrosinase-related protein 2* (*TYRP2*), which are required for melanin biosynthesis and transportation [2,25]. Upon UV irradiation, α-MSH derived from keratinocytes activates *MITF* and up-regulates the expression of its target genes via protein kinase A (PKA)-cAMP response element binding protein (CREB) signaling axis [25]. In addition, several transcription factors, such as SOX10 and LEF1, activate the transcriptional activity of MITF [26]. SOX10 (sex-determining region Y-box 10) canbind to the MITF promoter between −264 and −266 and increase MITF transcription [27]. LEF1 (lymphoid enhancer-binding factor 1) also transcriptionally cooperates with MITF as a non-DNA-binding activator for promoting MITF gene expression upon Wnt (wingless-type) signaling [28]. Post-translational modification of MITF, such as phosphorylation and acetylation, can regulate its own protein stability and activity [26]. Especially, phosphorylation of MITF at Ser73, where degradation-promoting PEST sequence is present, leads to proteasome-dependent MITF degradation in response to UV irradiation [17]. Proteasome-dependent MITF degradation is also caused by phosphorylation of MITF at Ser409 [18]. Phosphorylation of both Ser73 and Ser409 that promotes MITF degradation is dependent on the activation of ERK1/2 pathway [17,18]. In the present study, we found that ZER suppresses expression of MITF and its target genes, such as tyrosinase, TYRP1, and TYRP2 upon α-MSH stimulation, independent of PKA-CREB signaling pathway (Figure 6). Indeed, our results showed that ZER, but not arbutin and kojic acid, is sufficient to reduce α-MSH-induced tyrosinase mRNA and protein expression levels (Figure 2). These results demonstrate that ZER suppresses melanogenesis via the down-regulation of MITF-mediated transcription of melanogenic genes and their protein expression. Ubiquitin-mediated degradation of MITF is partly regulated by sustained extracellular signal-regulated kinases (ERK1/2) activation [6,7]. Our results showed that *Zingiber officinale* extract (ZO) and ZER increase ERK1/2 phosphorylation, and decrease melanin accumulation in B16F10 cells. Moreover, selective inhibitor of mitogen-activated protein kinase (MAPK), U0126, effectively restored melanin content, decreased by ZER, suggesting that ERK1/2 signaling is associated to the anti-melanogenic effect of *Zingiber officinale* (ZO) extract and zerumbone.

Decreased phosphorylation of ERK1/2 by ZER in hepatocellular carcinoma and U937 macrophage cells have been observed previously [29]. In addition, the ethanol extract of *Zingiber zerumbet* rhizomes has been shown to suppress ERK1/2 phosphorylation in diabetic retinas [30]. In contrast, in this study, we found that ZER increases phosphorylation of ERK1/2, but not MEK, in a dose-dependent manner (Figure 3A). Consistent with our result, a previous report has shown that 6-gingerol and 6-shogaol, which are the major active components of ginger, attenuate nerve growth factor (NGF)-induced ERK1/2 phosphorylation in mouse hippocampus [31]. Moreover, other experimental evidences have shown that ZER and 6-shogaol accelerate ERK1/2 phosphorylation in THP-1 monocytes and mouse B16F10 melanoma cells, respectively [24,32]. In addition, in mouse B16BL6 melanoma cells that were treated with isosakuranetin, a 4′-O-methylated flavonoid, a decreased phosphorylation of MITF and increased MITF stability has been observed through the suppression of ERK1/2 that subsequently stimulates melanogenesis [33]. Thus, we strongly suggest that ZER and ZO extract-induced ERK1/2 activation might be the reason for increased phosphorylation of MITF and its destabilization, which leads to the suppression of melanogenesis. Nevertheless, extensive investigation is necessary to address these controversies about *Zingiber* extracts and its components phosphorylate ERK1/2 differently in multiple types of cells or tissues. Because MEK is a major upstream kinase [34] that phosphorylates ERK1/2 upon oncogenic growth signaling, ZER was considered to alter activity of ERK1/2 upstream kinase as well. However, our results show that ZER does not affect MEK phosphorylation. Thus, there are two hypotheses for explaining molecular mechanism of ZER action. (1) ZER directly interacts and inhibits kinase activity of MEK via competitive or allosteric inhibitory mechanism, and (2) There are unknown signaling molecules that directly or indirectly get affected by ZER, and act as activators of ERK1/2. Interestingly, previous reports have shown that ZER causes oxidative stress through depletion of intracellular glutathione (GSH) and induction of intracellular reactive oxygen species (ROS) in colorectal and pancreatic cancer cells, respectively [35,36]. Moreover, it has also reported that increased intracellular ROS modulates ERK1/2 phosphorylation via suppression of dual-specific phosphatase 3 (DUSP3) by the oxidation of Cys-124 [37]. One possible hypothesis could be that increased oxidative stress and suppressed DUSP3 by ZER might be involved in ERK1/2 phosphorylation. In addition, Chen et al., has proposed that ZER attenuates intracellular nitric oxide (NO) accumulation by suppressing NF-κB and iNOS signaling pathway, which prevents mouse cornea from UVB-induced photokeratitis [38]. Nitric oxide (NO) is a melanogenesis-stimulating factor that is released from melanocytes and keratinocytes upon UV irradiation and proinflammatory cytokines [39,40]. This literature suggests the possibility that ZER attenuates α-MSH-induced melanogenesis through maintaining intracellular NO. Therefore, an extended study to demonstrate the molecular mechanism through which ZER activates ERK1/2 signaling pathway can provide scientific background for the development of skin-whitening cosmetics.

ZER has multiple biological functions, such as anti-inflammatory [41], anti-microbial [42], antioxidant [43], and anti-allergic [44]. Prolonged exposure to ultraviolet A (UVA) irradiation causes photoaging related dermatological disorders, such as wrinkles and skin cancer by excessive accumulation of reactive oxygen species (ROS) [2]. A previous report has shown that ZER exerts cytoprotection against UVA-irradiation-induced cellular damage in skin keratinocytes by increasing nuclear factor (erythroid-derived 2)-like 2 (Nrf2)-mediated antioxidants gene expression [1]. Our data suggests that ZER, as an active constituent of ZO extract, can be used to treat dermatological disorders, such as skin cancer, wrinkles, and hyperpigmentation, which are caused by UV irradiation. Although here we have shown the anti-melanogenic effect of ZER and ZO extract in B16F10 mouse and G361 human melanoma cells, their anti-melanogenesis activities must be further evaluated in human primary melanocytes prior to being considered in skin-whitening cosmetics.

## 4. Materials and Methods

### 4.1. Reagents and Antibodies

Antibodies against MITF (#12590), p-AKT^S473^ (#4060), p-CREB (#9398), p-ERK1/2 (#4370), ERK1/2 (#9102), p-MEK (#9154), MEK (#9122), and ERK1/2 inhibitor U0126 were purchased from Cell Signaling Technology (Danvers, MA, USA). Anti-Tyrosinase (sc-7833) and β-tubulin (sc-9104) were obtained from Santa Cruz Biotechnology (Dallas, TX, USA). Anti-TYRP2 (DCT, ab74073) was purchased from Abcam (Cambridge, UK). Zerumbone (Z3902), arbutin (A4256), kojic acid (K3125), α-MSH (M4135), and L-DOPA (333786) were purchased from Sigma-Aldrich (St. Louis, MO, USA). Stock solution of α-Melanocyte-stimulating hormone was prepared in phosphate buffered saline (PBS) prior to treatment. Recombinant human SCF was obtained from R&D systems (Minneapolis, MN, USA) and its stock solution (10 μM) was prepared in PBS. Stock solutions of zerumbone (20 mM), arbutin (1 M), and kojic acid (0.2 M) were prepared in dimethyl sulfoxide (DMSO). Lyophilized *Zingiber officinale* extract (035-061), isolated by 99% methanol, was obtained from Korea Plant Extract Bank (KPEB) (Daejeon, Korea) and Korea Research Institute of Bioscience and Biotechnology (KRIBB) (Daejeon, Korea). Stock solution of *Zingiber officinale* extract was prepared in DMSO prior to treatment.

### 4.2. Cell Culture and Cell Viability Assay

B16F10 (mouse melanoma), HaCaT (human keratinocyte) and G361 (human melanoma) cells were obtained from the Korean Cell Line Bank (Seoul, Korea) and cultured in Dulbecco’s modified Eagle’s medium (DMEM) supplemented with 10% fetal bovine serum (FBS) and 1% penicillin-streptomycin (P/S). Cells were incubated in a humidified atmosphere of 95% air and 5% CO_2_ at 37 °C. For cell viability assay, cells were incubated with different concentrations of zerumbone dissolved in dimethyl sulfoxide (DMSO) for 72 h. After incubation, cells were washed with cold phosphate buffered saline (PBS) and fixed with 4% paraformaldehyde for 15 min. After fixation, cells were incubated with 0.5% crystal violet staining solution for 20 min at room temperature. To measure optical density, the stained cells were treated with 1% sodium dodecyl sulfate (SDS) solution for 15 min at room temperature, and absorbance was measured at 570 nm (OD570) using an absorbance reader (BioTek, Winooski, VT, USA).

### 4.3. Immunoblotting and Immunoprecipitation

Immunoprecipitation was performed to detect whether endogenous MITF is phosphorylated at Ser73. 1 mg of cell lysates were incubated with 1 μg of anti-phospho-MITF antibody (pSer73; Sigma-Aldrich, St. Louis, MO, USA) for 16 h at 4 °C, followed by incubation with 20 μL of protein A/G-agarose beads (Santa Cruz Biotechnology, Dallas, TX, USA) for 3 h at 4 °C. Precipitated proteins were eluted in SDS sample buffer and then phosphorylated MITF (Ser73) protein was measured by immunoblotting using anti-p-MITF antibody (pSer73). Immunoblotting was performed, as previously described [4]. Briefly, total protein samples were prepared using lysis buffer, containing 1% NP-40 (Nonidet P-40), 150 mM NaCl, 50 mM Tris-HCl (pH 7.4), 10 mM NaF, and protease inhibitor cocktail. Sodium dodecyl sulfate-polyacrylamide gel electrophoresis (SDS-PAGE) was used to separate proteins in each sample based on their molecular weight. Separated proteins were then transferred onto polyvinylidene difluoride (PVDF) membrane (Millipore, Burlington, MA, USA). Membranes with transferred proteins were then incubated with primary antibodies (1:1000) and secondary antibodies (1:10,000) at 4 °C or room temperature. Chemiluminescent ECL Prime kit (GE healthcare, Pittsburgh, PA, USA) was used for visualizing protein expressions. 

### 4.4. Measurement of Intracellular and Extracellular Melanin Content

Intracellular and extracellular melanin content was measured and analyzed, as previously described [3]. Mouse melanogenic B16F10 cells were cultured with phenol-red free DMEM. Cells were then pre-treated with α-MSH (0.1 mM) for 1 h to promote melanogenic stimulation, and incubated with zerumbone for three days. After incubation, culture medium was transferred into fresh tubes and cultured cells were harvested and dissolved in 1 N NaOH containing 10% DMSO at 80 °C for 1 h. The melanin content of culture medium and cell extracts was measured at 475 nm (OD475) using absorbance reader. Melanin content was then normalized to the cellular protein concentration. 

### 4.5. Quantitative RT-PCR

Quantitative real time-PCR was performed as described previously [4]. Briefly, high capacity cDNA reverse transcription kit (Applied Biosystems, Waltham, MA, USA) and total RNA (2 μg) were used for cDNA synthesis. SYBR Green PCR Master MIX (Dynebio, Seongnam, Korea) was used for quantitative PCR. The sequence of the PCR primers 5′ and 3′ were as follows: TCAAGTTTCCAGAGACGGGT and CATCATCAGCCTGGAATCAA for MITF; ATAGGTGCATTGGCTTCTGG and TCTTCACCATGCTTTTGTGG for tyrosinase; CTCATCAAAGATGGCGTCTG and CTTCCTGAATGGGACCAATG for TYRP1.

### 4.6. Cellular Tyrosinase Activity Assay

Mouse melanogenic B16F10 cells were incubated with 0.1 mM of α-MSH in the absence or presence of zerumbone, arbutin, kojic acid, and *Zingiber officinale* (ZO) extract, as indicated. Cultured cells were then washed and lysed using cold PBS containing 1% Triton X-100, enzymatic activity of tyrosinase was measured using previously described methodology [4].

### 4.7. Statistical Analysis

Statistical significance was determined using unpaired Student’s t-test for two experimental comparisons and a two-way ANOVA with Tukey’s post-hoc test for multiple comparisons. Data are represented as means ± standard deviations (SD). *p* value < 0.05 was considered to be statistically significant.

## 5. Conclusions

The major findings of this study are that *Zingiber officinale* (ZO) extract and its active ingredient, zerumbone (ZER), (i) attenuates melanin accumulation upon α-MSH stimulation; and, (ii) decreases expression of melanogenesis-associated transcription factor, MITF, and its target genes by activating ERK1/2 independent of PKA-CREB signaling pathway (Figure 6). These results therefore suggest that *Zingiber officinale* (ZO) extract contained ZER, as an active ingredient, which would be useful in the development of both dermatological cosmetics and skin-whitening products.

## Abbreviations

α-MSH	Alpha-melanocyte stimulating hormone
L-DOPA	L-3,4-dihydroxyphenylalanine
MITF	Microphthalmia-associated transcription factor
TYRP1	Tyrosinase-related protein 1
TYRP2	Tyrosinase-related protein 2
CREB	cAMP response element binding protein
PKA	Protein Kinase A
ERK1/2	Extracellular signal-regulated kinase ½
